# Gender in Endocrine Diseases: Role of Sex Gonadal Hormones

**DOI:** 10.1155/2018/4847376

**Published:** 2018-10-21

**Authors:** R. Lauretta, M. Sansone, A. Sansone, F. Romanelli, M. Appetecchia

**Affiliations:** ^1^IRCCS Regina Elena National Cancer Institute, Endocrinology Unit, Rome, Italy; ^2^Department of Experimental Medicine, Section of Medical Pathophysiology, Food Science and Endocrinology, Sapienza Università di Roma, Rome, Italy

## Abstract

Gender- and sex- related differences represent a new frontier towards patient-tailored medicine, taking into account that theoretically every medical specialty can be influenced by both of them. Sex hormones define the differences between males and females, and the different endocrine environment promoted by estrogens, progesterone, testosterone, and their precursors might influence both human physiology and pathophysiology. With the term Gender we refer, instead, to behaviors, roles, expectations, and activities carried out by the individual in society. In other words, “gender” refers to a sociocultural sphere of the individual, whereas “sex” only defines the biological sex. In the last decade, increasing attention has been paid to understand the influence that gender can have on both the human physiology and pathogenesis of diseases. Even the clinical response to therapy may be influenced by sex hormones and gender, but further research is needed to investigate and clarify how they can affect the human pathophysiology. The path to a tailored medicine in which every patient is able to receive early diagnosis, risk assessments, and optimal treatments cannot exclude the importance of gender. In this review, we have focused our attention on the involvement of sex hormones and gender on different endocrine diseases.

## 1. Sex Hormones in Endocrine Gender-Related Differences

### 1.1. Introduction

Sex and gender intersect with other biological and social variables to produce between- or within-group differences [1]. Those factors may reveal subgroup differences among women and among men that would have been obscured by using only gender or sex as a variable. Accounting for differences in socioeconomic status, for example, may reveal unexpected differences between women and men that cannot be explained by gender or socioeconomic status alone, such as women of high socioeconomic status having health outcomes similar to those of men of low socioeconomic status [2]. Understanding how factors interrelate with sex or gender is important in explaining or predicting differences in health outcomes and determining user needs [3]. Among the medical disciplines, endocrinology is probably the one that most falls in issues concerning gender medicine. The hormones, in fact, determine sex, male or female, but it is now clear that the epidemiology, the clinical manifestations, the natural history of diseases, and the response to therapy can be very different in both sexes as well as because of the differences closely linked to the hormonal structure to the influence of social, economic, and cultural factors, which contribute to ensuring that men and women have important differences in health [4].

The interactions between sex and gender characteristics are supposed to affect molecular and cellular processes and clinical characteristics as well as health and disease outcomes [5]. Nevertheless, evidence concerning how they interact is scarce and requires a multidisciplinary approach. In this manuscript, we focused our attention on the sex and gender differences in endocrine pathophysiology. What makes females different from males is essentially represented by sex hormones and, in fact, the attention of scientific literature has been, and continues to be, an understanding of the biological mechanisms activated by sex hormones that underlie their pathophysiological diversities [6, 7]. Our attention has focused on the effects that not only sex as hormones but also gender can have in explaining differences in the human endocrine system. Conditions such as thyroid disease, diabetes mellitus, osteoporosis, GH/IGF I axis diseases, obesity, and sarcopenia clearly present gender differences [8–15]. Energy metabolism is also gender-specific, being greatly influenced by estrogen, both at rest and during exercise [16–18]. These hormones have also a considerable effect on the pathogenesis of autoimmune endocrine diseases, as suggested by their different prevalence, often significantly higher in women than in men [19].

Finally, there are sex and gender differences which also affect response to therapies, in terms of dose response, efficacy, and appearance of adverse events, although these aspects need to be further explored [20–28].

### 1.2. Gender-Related Differences in the Endocrine System

Gender-specific medicine is a complex and intriguing challenge for the future of all medical specialties. Hormones represent what makes females different from males. Generally, females and males have the same hormones (i.e., estrogens, progesterone, and testosterone), but their production sites, their blood concentrations, and their interactions with different organs, systems, and apparatus are different [29]. Males produce predominantly testosterone from the testes in a relatively constant daily amount according to a circadian profile. Small amounts of estrogens and progesterone are produced by the testes and the adrenal glands or are produced in the peripheral tissues, such as adipose tissue or liver, by the conversion of other precursor hormones [30]. In contrast, females mainly produce estrogens and progesterone from the ovaries in a cyclical pattern, while a small amount of testosterone (T) is produced by the ovaries and adrenal glands. The levels of female sexual steroids follow a specific and oscillating profile, due to a complex interaction between the pituitary gland and the ovary [31–33]. Female and male sexual steroids modulate in a different way, for example, the distribution of body fat mass and fluids, the maintenance of muscle and bone mass, the hepatic synthesis of numerous enzymes (cytochrome P450 family enzymes), the synthesis of triglycerides and HDL, and glucose metabolism. As a consequence, in addition to the typical differences of sex directly induced by hormones, we also have to consider the drug peripheral distribution and transformation pathways, especially hepatic and renal, which may be responsible for their reduced or increased efficacy as well as of the appearance of adverse reactions, for example due to their inappropriate doses or ways of administration. Moreover, socioeconomic and cultural contexts may represent additional confounding factors being able to influence the epidemiological characteristics of diseases, the approach, and the response to specific therapeutic treatments [9, 10, 13, 15].

### 1.3. Gender-Related Differences in Thyroid Disease

Thyroid diseases are 5 to 8 times more common in females than in males [34, 35]. Such data can be considered not only for clinical and/or subclinical hypothyroidism and hyperthyroidism and for nodular thyroid diseases but also for autoimmune conditions, such as Hashimoto's thyroiditis and Graves' disease [36, 37]. It can be hypothesized that female sex hormones (i.e., estrogens and progesterone) and their particular patterns may be involved in explaining the higher prevalence of thyroid diseases in females [37–39]. Differentiated thyroid carcinoma (DTC), the most common endocrine neoplasm, is also more common in women than in men, but evidence regarding gender-related differences is scant [40]. According to recent data, the age-adjusted incidence ratio of thyroid cancer in 2013 was 21.61/100,000 women and 7.26/100,000 men, for a female-to-male ratio of almost 3 : 1. However, because of differences between sexes in thyroid cancer peak age incidence, the female-to-male ratio is higher during the reproductive period (4.1 : 1 at ages 20–49 years) and steadily decreases with advancing age (1.38 : 1 at ages ≥ 75 years). Furthermore, females have a better survival rate than age-matched males. Therefore, thyroid cancer is more common in females but is more aggressive in males [41, 42]. Pathophysiological reasons explaining this difference are unknown, but it has been proposed that estrogens may play a fundamental role. This hypothesis is supported by evidence that thyroid cancer has a higher incidence in fertile women [43]. A causal role for the number of children, age at first pregnancy, age at onset of menopause, and hysterectomy has also been suggested [44]. According to a recent meta-analysis, women with children have an increased risk of thyroid cancer compared to nulliparous women (Relative Risk 1.09, CI 1.03–1.15), but a linear relationship between the number of children and the increased risk has not been proven [40]. Nevertheless, the recent guidelines published by the American Association of Clinical Endocrinologists and the American College of Endocrinologists in 2015 suggest that clinical trials do not support the role of estrogens as a risk factor for the development of thyroid cancer at present [45]. Female sex, together with the absence of lymph node metastases, and the American Thyroid Association (ATA) pediatric risk stratification system remained factors related to better outcomes in pediatric DTC, even in longer periods of observation (i.e., 32 years). Furthermore, girls with no lymph node metastasis at diagnosis and those classified as low risk by the ATA pediatric risk stratification system were more likely to have no evidence of disease within the first year compared to boys [46]. Regarding the therapeutic aspects, gender does not affect the function of salivary glands in patients affected by thyroid cancer undergoing first attempt of radioactive iodine therapy [47].

### 1.4. Gender-Related Differences and Diabetes Mellitus

Recently, it has been observed that diabetes mellitus may also have some gender-specific peculiarities; some data highlight that women have longer-term illness and higher body mass index (BMI) compared to men [14]. In women, diabetes mellitus appears to be less controlled considering each metabolic parameter. Italian data from annals published by Associazione Medici Diabetologi (AMD), the Italian association of diabetologists, showed that diabetic women had 14% higher chance of having HbA1c > 9% regardless of insulin therapy, 42% more likely to have low-density lipoprotein (LDL) cholesterol > 130 mg/dL irrespective of statin therapy, and 50% greater chance of having BMI > 30 kg/m^2^ [48, 49]. These data seem to be partially confirmed in type 1 diabetic patients, in whom women showed worse metabolic control and men had higher blood pressure [13]. Furthermore, diabetic women, regardless of menopausal state, present significantly higher risk of ischemic cardiomyopathy than diabetic men. Diabetic women also have a worse prognosis after myocardial infarction and a higher mortality rate from cardiovascular disease than diabetic men [50, 51]. A Canadian study showed that long-term statin therapy reduces total and cardiovascular mortality after myocardial infarction, and this effect is pronounced over time in both sexes. However, this risk reduction is lower in women than in men, suggesting a gender-specific model of therapy response [25]. Regarding the metabolic aspect, women show a different behavior in their insulin response compared to men. In fact, the susceptibility to develop insulin resistance and the insulin response to stimuli that physiologically improve or compromise insulin sensitivity are different in the two sexes [52]. Women show a tendency to have lower insulin sensitivity than their male counterpart but increase their insulin response to maintain normoglycemia (Table 1) [53, 54]. It can suggest that these differences in insulin action may explain that in prediabetic state women are more prone to develop impaired glucose tolerance whereas their male counterparts are more susceptible to develop impaired fasting glycemia [55, 56]. This gender-related physiology may underline the different effects showed by a combined therapy with exenatide and metformin which induced better therapeutic results in women compared with men [57]. Interestingly, the difference in sex affects the prevalence of diabetes that is reversed according to the stage of reproductive life. There are more diabetic men before the age of puberty, while there are more diabetic women after the age of menopause and in old age [55]. Recent evidence confirms higher prevalence of T2DM in adolescent females than males associated with a greater insulin resistance in girls than in boys during puberty. Beyond puberty, T2DM is more common in middle aged men; different patterns of fat accumulation providing with a greater subcutaneous fat presence in women along with better insulin sensitivity may represent possible reasons [58]. The role of menopausal estrogen deficiency in the increased risk of type 2 diabetes mellitus in menopausal women has been extensively studied. It should be considered that estrogens affect positively glucose homeostasis within a physiological window and any change outside the physiological range, such as menopause or oral contraceptives, represents a risk factor for insulin resistance [55, 56]. In diabetes mellitus, women are at higher risk of experiencing hypoglycemia using insulin and for urinary tract and genital infection using gliflozin drugs. As a result of the use of thiazolidinediones, the risk of bone fractures in postmenopausal women increases [15]. Finally, recent studies have observed a marked influence of socioeconomic and psychosocial aspects on glycemic health control and a significant association between sociodemographic profile and absolute control of T1DM risk factors [59, 60]. Furthermore, T2DM women and men with lower income and education level show poor food choice revealing higher carbohydrate and lower fat intake. Considering the sex-related difference in insulin sensitivity, it is clear how sociodemographic aspects may interact with biology [61]. This association has been observed also in pregnant woman [62]. Even in the prevention and management of gestational diabetes mellitus, maternal income and education may have a strong impact [63]. Keeping in mind that the term “gender” refers to social and psychological differences between men and women, further studies are warranted to clarify the influence of gender on glycemic health and the interactions with biological distinctions [4].

### 1.5. Gender-Related Differences and Osteoporosis

A large part of clinical evidence is based on trials on male subjects, creating the so-called male-bias evidence-based medicine. Nevertheless, scientific studies on osteoporosis represent an exception to male-bias evidence-based medicine. In fact, osteoporosis has always been considered a typical female disease, although it is also common in males. As a consequence, osteoporosis is most commonly investigated in women, especially after menopause, and it is rarely considered in men, who also present significant risk factors [64]. Indeed, a lower proportion of men at high risk of fracture are treated than women at high risk [65]. Men also tend to have worse outcomes after fracture than women; they are twice as likely to die after hip fracture as women [66].

Interestingly, the Epidemiologic Study on the Prevalence of Osteoporosis (ESOPO), the main Italian epidemiologic study on osteoporosis, was conducted on 11,011 females and 4981 males and showed that in females the prevalence of osteoporosis was about 18.5%, while in males it was about 10%. Similarly, the prevalence of osteopenia was 44.7% and 36%, respectively. The presence of bone fracture was confirmed in 17.6% of females and in 17.5% of males. Thereafter, mortality was 2–3 times higher in males suffering with femur fracture than in females. These data suggest that bone health and status should be carefully evaluated even in elderly males [67]. The physiopathology of osteoporosis is clearly sex-specific. Males tend to have higher bone mineral density and bone content and reach it at an older age than females, while females tend to lose bone density at a younger age and at a faster rate than males and also have higher bone reabsorption markers [68]. Later in life, the production of sex hormones decreases earlier and more markedly in females. This aspect can be the basis of the presence of fractures about 5–10 years earlier in females than in males [69]. Indeed, estrogens play a crucial role in bone health in both sexes, and their deficiency is supposed to be the main cause of bone loss in postmenopausal females and in elderly males, in particular for cortical bone [70]. Estrogens inhibit generation and activity of osteoclasts through an upregulation of osteoprotegerin, decrease T-cell activation and consequently also interferon-*γ* release by T-cells, and increase intestinal calcium absorption [71, 72]. In females, estrogen decline is abrupt at the beginning of the menopausal period, while in males the decline in T and, consequently, in estrogen, is low and constant with aging, so it is clear how sex differences in osteoporosis exist. An increase in both bone formation markers and bone reabsorption markers have been observed in postmenopausal females, suggesting an increase in the rate of bone remodeling as confirmed by histomorphometry. In elderly males, biochemical markers of bone degradation seem to increase but bone formation markers appear to be stable or decreased, therefore suggesting a low bone remodeling rate (Table 1) [8]. Furthermore, an alteration of the inflammatory state of the bone has been demonstrated to be more pronounced in postmenopausal females than in older males, thus negatively affecting bone health [73, 74].

According to the underlying causes, osteoporosis may be primary or secondary. Prevalence of secondary osteoporosis in females reaches up to 20–40% of cases, while this value rises to 65% in males [75]. Scientific studies on osteoporosis therapy have mostly focused their attention on females, then considering their results applicable to males. However, it seems that females are more prone to suffer from side effects associated with bisphosphonates. The higher relative cases of atypical bone fractures in women than in men are not entirely related to an increased use of bisphosphonates but also to sex per se, which should be considered a risk factor for atypical fracture [76]. Some oncological diseases (breast and prostate cancer) can induce modification of the bone metabolism, also aggravated by the use of anticancer therapies both in women and in men. Hormone therapy (aromatase inhibitors such as anastrozole, letrozole and exemestane, LHRH analogs, bicalutamide, and abiraterone) and chemotherapies can induce a greater bone resorption compared to its synthesis, creating a net effect of loss of bone mass, a reduction of resistance and a consequent increase in bone fractures in both women and men, even in the absence of trauma. Global bone health can also be compromised by high doses of cortisone associated with certain treatments and pathological changes related to oncology in itself (early menopause in women and hypogonadism in men).

### 1.6. Gender-Related Differences and the Growth Hormone/Insulin Growth Factor 1 Axis

The clinical evidence supports the effects of estrogens on the growth hormone (GH)/insulin growth factor 1 (IGF-1) axis [77]. In fact, several studies have shown that estrogens inhibit GH-stimulated liver production of IGF-1 [77, 78]. In turn, GH levels rise to overcome the inhibitory effects of estrogen. It has been observed that the levels of GH are higher in females than in males, and they fluctuate according to the phase of the menstrual cycle and depending on the menopausal state [79]. Moreover, during the first trimester of pregnancy, estrogen levels increase and consequently IGF-1 levels decrease in the absence of any change in GH levels. IGF-1 levels increase from the beginning of the second quarter due to the gradual increase in placental GH [80]. The effect of estrogens on the GH/IGF-1 axis is also noteworthy even in pathologies characterized by deficiency or excess of GH.

Females suffering from GH deficiency require a much higher dose of recombinant GH (rhGH) than males. Women taking oral estrogens need a higher dose of rhGH than those taking transdermal estrogens [81, 82]. It can be hypothesized that the inhibitory action of oral estrogens on the metabolic effect of GH is mediated by stimulation of cytokine 2 suppressor expression (SOCS-2), which in turn inhibits the phosphorylation of Janus kinase 2 (JAK2), a key passage in the signaling path JAK2/signal transducer and activator of transcription 5 (STAT5) activated by GH. The inhibition of enzymatic function of JAK prevents GH from exerting its metabolic effects, including the hepatic synthesis of IGF-1 [83]. Indeed, stimulated JAK2 adds a phosphate group to specific tyrosine residues on the cytoplasmic domain of the GH receptor. Therefore, using its Src homology 2 (SH2) domain, STAT5 binds to these phosphorylated tyrosine residues [84]. The bound STAT5 is phosphorylated by JAK2 to specific tyrosine residues and is ready to form homodimers or heterodimers to act as a transcription factor (Table 1) [11]. Females suffering from acromegaly show lower IGF-1 levels than males who suffer from the same condition. It is interesting to highlight that in some specific acromegalic females, IGF-1 levels decrease during the first trimester of pregnancy [85]. A possible explanation could be the physiological increase in estrogen levels and their subsequent inhibition of IGF-1 production in the liver [84]. This mechanism can be considered as a possible reason for the improvement of the clinical conditions of acromegalic females during this period [84]. Acromegaly has also shown some clinical differences between the sexes, as specific metabolic alterations of the acromegaly are sex-specific. Acromegalic females are more prone to suffer from insulin resistance and metabolic syndrome than males, even in the absence of significant differences in blood glucose and/or glycated hemoglobin (HbA1c). In addition, a higher prevalence of metabolic syndrome, visceral obesity, and diabetes mellitus was observed in postmenopausal females compared to premenopausal females and males [12]. It is interesting to note that the administration of rhGH often leads to hypothyroidism through both central and peripheral mechanisms; in particular, rhGH appears to decrease the TSH level by increasing IGF-1 [86]. In fact, IGF-1 seems to be involved in the direct stimulation of somatostatin mRNA synthesis and, in turn, somatostatin inhibits TSH secretion [87]. This process has not always been observed in females, and it seems that gonadal hormones play a fundamental role due to their inhibition of IGF-1 secretion, as mentioned above, and their downregulation of the thyroid somatostatin receptor (SSTR) by estrogen. SSTRs 1, 3, 4, and 5 are highly expressed in normal thyroid tissue, and estrogen has a differential effect on distinct SSTs, subregulating the expression of SSTRs 1 and 5 (Table 1) [11].

### 1.7. Gender-Related Differences and Obesity

Females and males showed marked differences in the prevalence of obesity, in fat deposition patterns, and in fat metabolism. Females generally have a higher percentage of fat mass and are more likely to deposit fat subcutaneously and on their lower extremities while men are more likely to deposit visceral fat in the abdominal region [88]. Adipose tissue increases with puberty and early pregnancy, suggesting that gonadal steroids can influence body fat. Following menopause-induced estrogen loss, a shift towards visceral adiposity occurs, which is sensitive to estrogen therapy [89]. These facts highlight the importance of estrogens in subcutaneous fat accumulation. At cellular level, estrogen function is mediated by alpha (ER*α*) and beta (ER*β*) receptors although recent research observed nongenomic and rapid effects of steroid hormones throughout cytosolic or plasma membrane-associated receptors. Both ER*α* and ER*β* are expressed in subcutaneous and visceral adipose tissue; however, it seems that ER*α* plays a pivotal role in sexual dimorphism of fat distribution. Female and male mice that lack ER*α* have visceral obesity with severe insulin resistance. Furthermore, estrogens seem to promote and maintain the typical female type of fat distribution by affecting lipolysis, which is controlled in humans primarily by the action of *β*-adrenergic receptors (lipolytic) and *α*2A-adrenergic receptors (antilipolytic). Estrogens increase the number of antilipolytic *α*2A-adrenergic receptors in subcutaneous adipocytes; in contrast, no effect of estrogens on *α*2A-adrenergic receptor mRNA expression was observed in adipocytes from the intra-abdominal fat depot where a high *α*2A/*β* ratio is present [90]. In premenopausal women, *α*2A to *β*1–2-adrenergic receptor ratio is increased in subcutaneous fat tissue compared to men and to postmenopausal women thus decreasing the lipolytic response to adrenergic and noradrenergic stimuli. This balance of adrenergic receptors is reversed in the visceral depot of premenopausal women, favoring lipolysis of visceral fat and it accounts for deposition of fat in subcutaneous adipocytes in premenopausal women. In postmenopausal females, adrenergic receptor ratio is reversed thus potentially explaining the preferential accumulation of fat in the visceral depot. Therefore, at the adipocyte levels, estrogens and their receptors may have the capacity to increase the accumulation of fat cells in the subcutaneous deposit and to inhibit it in the visceral deposit [91].

This pattern of fat accrual affords protection from the negative consequences associated with obesity and the metabolic syndrome in premenopausal females. However, after menopause, the decrease of estrogen secretion leads to fat deposition and accrual shift in favor of the visceral depot. This shift is accompanied by a parallel increase in metabolic risk similar to that seen in males. Estrogen appears to protect against obesity also through the suppression of appetite, as observed during the periovulatory phase of the menstrual cycle, and by increasing energy expenditure [91]. T exerts its antiobesity effect by activation of the androgen receptor (AR) pathway on mesenchymal stem cells, suppressing the adipogenic line cells and favoring the myogenic line. Furthermore, T increases lipolysis and the number of *β*-adrenergic receptors on the membranes of adipocytes and inhibits triglyceride uptake and lipoprotein lipase activity. Nevertheless, in females, hyperandrogenism positively correlated with visceral fat, waist circumferences, and insulin resistance. Androgen excess may induce these effects through both central and peripheral mechanisms. Failure to activate leptin with consequent blockage of brown adipose tissue thermogenesis and reduced expression of hypothalamic proopiomelanocortin may represent important central control mechanisms. Peripherally, the interaction with estradiol may explain the different effects of T on women metabolism [92]. Additionally, cross-sex hormonal therapy of male-to-female transsexuals increases the amount of subcutaneous adipose tissue accrual relative to intra-abdominal adipose tissue whereas more masculine body fat distribution with a lower hip circumference has been observed in trans males [93].

Finally, a sex-specific fat deposition pattern represents a physiological condition in which sex hormones play a pivotal role. Nevertheless, in both sexes, the presence of normal sex hormone levels is protective against obesity, and a tendency to increase central obesity is observed with a decrease in sex steroid hormones, as it happens with old age or gonadectomy [94].

Estrogens also affect fuel metabolism by reducing postprandial fatty acid oxidation, leading to an increase in body fat which may account for the increased fat mass observed in women compared to men and the increased fat early in pregnancy (Table 1) [95]. Interestingly, O'Sullivan et al. showed that basal lipid oxidation was reduced in pregnant and nonpregnant women compared to postmenopausal women, and postprandial lipid oxidation was reduced in pregnancy compared to nonpregnant healthy women, who in turn have lower postprandial lipid oxidation than postmenopausal women (Table 1) [96]. A possible explanation for this efficient fat storage of energy in female puberty and in early pregnancy is the obvious biological advantage in preparation for fertility, fetal development, and lactation [95]. Otherwise, women show a greater reliance on fat oxidation than men during submaximal exercise, showing a higher maximum fat oxidation rate and a fat oxidation curve which tends to be shifted toward higher exercise intensities [97]. It seems likely that both genomic and nongenomic actions of estrogens may play a role in explaining these observations. In particular, estrogens mainly act through estrogen receptor-alpha in the skeletal muscle to stimulate the genomic expression of proteins to increase the availability of long chain fatty acids (LCFA) improving adipocyte lipolysis and increasing intramyocellular lipid storage. Following on, estrogens affect fuel metabolism during exercise by nongenomic means to increase the activation of 5′ adenosine monophosphate-activated protein kinase (AMPK) [16, 98].

### 1.8. Gender-Related Differences in Sarcopenia

Sarcopenia is an age-related syndrome defined by the loss of muscle mass and strength and/or performance, often associated with chronic diseases, obesity, and prolonged immobilization. However, it also represents a physiological condition of aging [99, 100]. The etiology of sarcopenia is multifactorial but still poorly understood. A decrease of anabolic hormones plays a role in the development and in the maintenance of sarcopenia [101]. In particular, the decrease in T appears to be crucial in elderly males, and the administration of T in hypogonadal subjects may be extremely helpful in limiting the loss of muscle mass and strength (Table 2). Indeed, T administration is able to significantly increase muscle mass and decrease fat mass also in eugonadal males, even in total absence of any training stimulus [102–104]. T is also of paramount importance in supporting adaptation to strength training. Indeed, in evaluating sarcopenia, three aspects should be carefully evaluated: physical exercise, nutrition, and hormonal homeostasis [101]. In particular, strength training represents the form of physical exercise which has the greater positive impact in limiting loss of muscle mass. However, it has been observed that the adaptive process to strength training requires adequate serum level of T [105]. Therefore, in the presence of overt hypogonadism, strength training results to be almost useless and ineffective in the therapy of sarcopenia (Table 2). Aside these effects, the positive effects of T on erythropoiesis and on mood should be also considered as they may support elderly men to follow a consistent training schedule [106–108].

In postmenopausal women, evidence regarding the effect of abrupt decrease of estrogens on muscle mass and strength and eventually the impact of hormonal replacement therapy (HRT) is scarce. During menopause, females show a marked decrease in muscle mass and strength, while in males this loss is constant and takes place more slowly. However, this is not shown in women undergoing HRT [109]. A meta-analysis showed that strength was significantly greater in women on HRT. The effect sizes (ESs) were calculated as the standardized mean difference and amounted to 0.23, equating to women on HRT being ∼5% stronger. Nevertheless, effect sizes tended to be greater (∼0.45) when only randomized; controlled trials were considered or when strength was normalized for muscle size, indicating that estrogens affect positively muscle strength and contractile properties of muscle tissue [110]. Estrogens appear to act via several mechanisms, such as increased levels of proanabolic factors, reduced systemic and muscle inflammation, decreased muscle damage, and augmented postexercise muscle satellite cell activation and proliferation. Furthermore, estrogens also seem to improve intrinsic contractile muscle function altering myosin functions, as reported by increased strength normalized to muscle size (Table 1) [109, 111]. A recent study with monozygotic twin pairs showed that thigh muscle cross-sectional area tended to be larger, relative muscle area greater, and relative fat area smaller in HRT users than in their sisters. In particular, tibolone administration, a tissue-specific compound with estrogenic, progestogenic, and weak androgenic activities [112], increased muscle cross-sectional area [113]. Tibolone showed promising effects, increasing significantly handgrip strength compared to placebo in postmenopausal women and improving markedly isometric knee extension strength, adjusted for BMI [114]. In a cross-sectional study, mean knee extensor strength was higher in women taking tibolone or estrogen compared to no HRT [115]. Following on, tibolone seems to affect body composition, increasing lean mass and decreasing total body fat mass [112, 116–118]. Thus, the lower rate of falling in the tibolone group observed by Cummings et al. might reflect an androgenic effect on muscular function [119].

### 1.9. Gender-Related Differences in Drug Response

Most scientific studies on drug response have been performed on male subjects and the results have been considered valid also for females, assuming that sex did not affect the outcome (Yentl syndrome) [120, 121]. However, it should be noted that in 2005 eight out of ten prescription drugs were withdrawn from the US market because of women's health issues [122]. Recently, the NHS and Medical Research Council have evaluated the causes and effects of women's sociodemographic exclusions from clinical trials. Therefore, the use of statins and nonsteroidal anti-inflammatory drugs (NSAIDs) has been investigated. These drugs demonstrated a marked difference in the sex of subjects included in the trials. Studies on NSAIDs have reflected the population in which they were used, while those for statins did not and only 16% of women were included in trials compared with 45% who were using statins [123]. The therapeutic response may be different between genders, and even if evidence is far from being conclusive, the existing data have to be considered and evaluated [124]. Indeed, it is well known that cytochrome expression may be a gender-specific mechanism involved in difference in drug metabolism [125, 126]. Concerning lipid-lowering drugs, it has been observed that women on atorvastatin had more side effects (i.e., increased liver enzymes and myalgia) than men. However, atorvastatin and rosuvastatin seemed to have similar efficacy in both sexes [26]. On the contrary, fenofibrate improved lipid profile more in women than in men and reduced cardiovascular events by 30% in women and 13% in men [27, 127]. In evaluating gender differences, it should be considered that patients with lower income are more likely to use generic drugs. The presence of different excipients may affect drug response therapy according to sex [128].

## 2. Conclusions

Sex- and gender-specific differences can be observed in several endocrine diseases, but the majority of these aspects have not been carefully assessed so far. However, evidence in the scientific literature holds that sex and gender should always be considered in every element of the disease, from the causes to the treatment.

## Figures and Tables

**Table 1:** Endocrine roles of estrogens in the human body.

Thyroid	Increase TBG; decrease free fraction of thyroxine [129]; downregulation of the thyroid somatostatin receptor (SSTR) [11, 129]

Glucose metabolism	Increase insulin sensitivity; protect pancreatic *β*-cells [130]

Bone	Inhibit generation and activity of osteoclasts; upregulation of osteoprotegerin; decrease T cell activation; decrease IFN-*γ* release by T cells; increase intestinal calcium absorption [8]

Muscle	Increase levels of proanabolic factors; reduce muscle inflammation; decrease muscle damage; increase postexercise muscle satellite cell activation and proliferation; increase intrinsic contractile muscle function [109, 110]

GH/IGF-1 axis	Decrease hepatic IGF-1 production; downregulation of the thyroid somatostatin receptor (SSTR) [11]

Adipose tissue	Increase gynoid fat deposition [88]; decrease postprandial fatty acid oxidation [95, 96]; increase fat oxidation during submaximal exercise [16, 98]; decrease energy intake; increase energy expenditure; reduce tissue inflammation [130]

**Table 2:** Endocrine roles of T in the human body.

Sexual function	Increase libido and erectile function [131]

Glucose metabolism	Increased insulin sensitivity [132, 133]

Bone	Increased bone mineral density; reduction in bone resorption markers Decrease of osteoblast apoptosis rate Stimulation of osteoprogenitor cell proliferation and of differentiation of mature osteoblasts Decrease of osteoclast formation and bone resorption through an increased production of osteoprotegerin [134–136]

Muscle	Increase in both cross-sectional area and myonuclei number; increased muscle fiber area via hyperplasia and hypertrophy; increased muscle strength [101]

Haematopoietic system	Stimulation of erythropoiesis directly and erythropoietin synthesis in the kidney Promotion of erythropoietic stem cell differentiation and increased sensitivity of erythroid progenitors to erythropoietin [137, 138]

Adipose tissue	Commitment of pluripotent mesenchymal cells into myogenic lineage; inhibition of commitment of pluripotent mesenchymal cells into adipocyte lineage and inhibition of differentiation of subcutaneous abdominal preadipocytes into adipocytes. Net decrease in fat mass [101]
